# External Ulcerative Ano-Vulvitis of Cattle*Read at the Thirty-ninth Annual Meeting of the American Veterinary Medical Association, Minneapolis, Minnesota, September 2, 3, 4, and 5, 1902.

**Published:** 1902-09

**Authors:** John J. Repp

**Affiliations:** Veterinarian to the Experiment Station, Ames, Iowa


### EXTERNAL ULCERATIVE ANO-VULVITIS OF CATTLE. A PRELIMINARY REPORT.*

By John J. Repp, V.M.D.,
VETERINARIAN TO THE EXPERIMENT STATION, AMES, IOWA.

*Terminology.* The name first applied to the disease under consideration so far as I have been able to learn was "contagious vulvitis of cattle." This name was given by S. Stewart[1] in a verbal report before the Missouri Valley Veterinary Association, February 9, 1898. The name next put upon record was "infectious ulcer of the vulva," a name given by C. Miller[2] in a paper read before the Missouri Veterinary Medical Association, October 3 and 4, 1900.

In November, 1901, while investigating this disease in an outbreak which occurred in the practice of S. T. Miller, he told me that he intended to contribute a paper upon the disease for the next meeting of the Iowa State Veterinary Medical Association, to be held February 11 and 12, 1902. I expressed to him the opinion that the names already given to the disease were not appropriate, an opinion in which he readily concurred. He then asked me to substitute a name which he might adopt in his paper. After further study of the disease I submitted the name which appears in the title of this paper. I did this with a full realization of the enormity of the offense of multiplying synonyms and confusing the terminology of a disease. I believe, however, that if a disease has been badly named it is the duty of some one to find a name which is of logical origin and which will be likely to meet with general approval.

The objection to the names already given was twofold. In using the word contagious in the one name and the word infectious in the other, the name is made to assume too much. A dis-

* Read at the Thirty-ninth Annual Meeting of the American Veterinary Medical Association, Minneapolis, Minnesota, September 2, 3, 4, and 5, 1902.

ease should not be designated infectious or contagious until there is fair proof that it is infectious or contagious. Such proof is lacking in case of this disease. The names were not comprehensive enough. They gave but a partial idea of the nature of the process. A name should be as fully descriptive of a disease as is compatible with the enforced brevity of a name as applied to a disease. Since adopting the name of my choice for this disease the *Seventeenth Annual Report of the Bureau of Animal Industry* has been published. On page 28 of that volume reference is made to a disease, which I assume to be the same as the one now under discussion, as gangrenous vulvitis. This name is unsuitable, because the process is not gangrenous and because the name is not comprehensive enough.

In defence of the name which I have applied to the disease I would say that the disease is primarily and essentially inflammatory, and the name ano-vulvitis sets forth this characteristic and at the same time indicates the location of the morbid process. As the inflammation is ulcerative in character—*i. e.*, characterized almost from the beginning by erosion and superficial loss of tissue, it is best defined by the adjective ulcerative. As the disease has its inception, and in mild cases its localization, upon the external surface of the labiæ of the vulva it is very appropriately described by the term external. If the mucous membrane of the vulva, which may be considered the internal part, is invaded at all, the invasion is secondary to the skin lesions, and occurs only in severe cases.

In applying a new name to this disease I have not been unmindful of the tendency, and in a large measure the accomplished purpose, of medical men to refer all diseases of the external genitals to the domain of dermatoses and to describe each affection under the name of some skin disease which may occur anywhere upon the body. However, the fact that the disease now under consideration extends deeply into the tissues, far beyond the skin, entitles it to a place in nomenclature along with such pathological entities as gonorrhœa, chancroid and syphilis. One would not be justified in essaying a new name for a disease unless he had first made a diligent effort to identify that disease with one already well known, either in human or in veterinary pathology. It is plainly evident that there is no well-defined disease in animals with which this could be identified. In the field of human pathology I have attempted to identify this disease with noma of the vulva, herpes progenitalis and chancroid, the only diseases of human beings which it even remotely resembles, but without success. Syphilis must, of course, be thought of only to be at once dismissed from consideration. The resemblance to chancroid is in many respects striking, but the difference between them is quite distinct. In order not to prolong this discussion beyond reasonable length, I refrain from a presentation of the distinguishing features of these various diseases and the one under discussion, and, instead, refer those interested to recent standard works of genito-urinary diseases and gynecology.

If any one here, now or at any other time, can supply another name and furnish convincing evidence that it is preferable to the one I propose, I will be very willing to abandon my name for his.

*History.* The earliest date at which a report of this disease was made to a veterinary body so far as I have been able to learn was February 9, 1898, at which time S. Stewart made a verbal report to the Missouri Valley Veterinary Association. The Secretary of that Association reports[3] Stewart as saying that a large herd of heifers, originally from about Trinidad, Colorado, was brought to Kansas City and distributed in small bunches among farmers, and that all of these heifers became affected. I assume that these heifers were afflicted with the disease now under consideration.

C. Miller[4] reports that in February, 1898, he observed this disease in two herds of young cattle near Ottumwa, Iowa.

Steddom[5] reports that on February 17, 1898, he began the investigation of what was doubtless an outbreak of this disease in Marshall County, Kansas. He visited four herds in which the disease existed. He was able to trace the outbreak in one herd back as far as December 25, 1897. The owner suspected that one cow had had the disease and had already recovered when he brought the herd to his farm December 18, 1897. This is the earliest outbreak of this disease that I have any information about.

Parker[6] reports that in December, 1899, Steddom investigated an outbreak of this disease at Westmoreland, Kansas.

S. H. Johnston[7] has informed me that in the winter of 1899 he met with an outbreak at Lake City, Iowa.

In January, 1900, I had my first experience with this disease in an outbreak which occurred in a herd at Harlan, Iowa, in the practice of D. H. Miller.

In 1900 or 1901 a disease which, from the brief description given, I would suppose to have been ulcerative ano-vulvitis was reported from Ohio.[8]

*The Seventeenth Annual Report of the Bureau of Animal Industry* contains, on page 28, the following statement by Salmon: "Two outbreaks of gangrenous vulvitis in cattle were reported during the fiscal year, and specimens forwarded . . . for examination."

In November, 1901, I investigated outbreaks in several herds at Shelby, Iowa, in the practice of S. T. Miller. These outbreaks have already been reported upon by Miller[9] in a paper read before the Iowa State Veterinary Medical Association, February 12, 1902.

In November, 1901, an outbreak occurred in a small herd near Ames, Iowa.

In December, 1901, an outbreak occurred in a herd at Humboldt, Iowa, in the practice of J. Nicholson.[10]

This is as much of the history of the disease that I have been able to procure. I would not venture to assert that this is a new disease. It is certain, however, that it has not as yet been adequately studied and described. It is not improbable that the disease has been known to veterinarians and laymen for a long time, but no one has been actuated to make mention of it in writing until within the last five years, and then only very briefly.

*Kinds of Animals Affected.* So far as I have been able to learn external ulcerative ano-vulvitis is circumscribed to the bovine species. Females are by far the most frequently affected. In one herd of twenty-five cattle, one to six years of age, which I inspected, in which were twenty-one females and four males, all of the females were affected, but none of the males. In another herd which I inspected there were in one bunch thirty-two calves, eight to ten months old, of which fifteen were heifers and seventeen steers. All of the heifers and only three of the steers became affected. In another bunch were eight young calves, half male and half female. The females were all diseased, but the males did not become involved. In another herd which came under my notice fifteen heifers became affected, and after a considerable time two steers acquired the disease. Other steers in the same lot escaped. In still another herd in a large feed yard there were being fed about fifty cows and seventy-five steers. All the cows were affected, but only six steers became involved. Steddom inspected one herd of twenty-five, seventeen steers and eight heifers, in which all of the heifers but none of the steers became affected.

To show that there are some exceptions to this I may state that in another herd eight heifers and thirteen steers were being fattened in the same yard, and all acquired the disease.

In several instances ulcerative ano-vulvitis has been observed in spayed heifers.

In respect to age it may be stated that although it seems rather more apt to affect young cattle, there is very little difference in susceptibility on account of age. I have seen it in cattle ranging from two weeks to ten years of age. In one herd of thirty-two head a very aged cow was the only one which escaped. In another case a bunch of twenty-two adult cows being fattened in a yard were affected, and later a number of calves in an adjoining yard became involved. I have never observed the disease in an adult bull.

*Season when the Disease Occurs.* Ulcerative ano-vulvitis appears to be a disease of cold weather. I have not seen or heard of its eruption earlier than October 15th or later than March 15th.

*Etiology.* I shall refrain from an extended discussion upon this important part of the subject, for any statement I could make would be purely hypothetic. I regret that my opportunities of time and place have not enabled me to make the study in this connection which is so very essential to a complete knowledge of the disease. The cause of the disease remains thus far entirely unknown. It may be stated, however, that in every outbreak which I have studied the affected cattle were kept in filthy yards which had been used jointly by cattle and hogs for eighteen to twenty-five years. Some of these yards are poorly drained, and at times when the temperature is high enough to cause a thaw they become veritable mud-and-manure-holes. Cattle have not been known to acquire the disease while at pasture. There is no evidence that the disease has any causal relation to coitus.

*Is External Ulcerative Ano-vulvitis Contagious?* My observations and those of others indicate that it is probably not contagious. On one farm where I made observations there were eight calves kept in an orchard, where the ground was clean, adjoining a stable-yard in which were twenty-five cattle, all but four of which were affected. Only a fence separated the two bunches of cattle, yet none of the eight calves became in the least affected. In another outbreak the owner traded a cow affected with ulcerative ano-vulvitis to a neighbor for a cow which was free from the disease. None of the neighbor's herd into which the diseased cow went became affected. Incidentally, it may be stated that the healthy cow that went into the diseased herd became affected within a week. S. T. Miller[11] informs me that in one instance which came under his observation a bull from a healthy herd broke through a fence into a neighbor's herd which was suffering from ano-vulvitis, served a diseased cow there, then returned to his own herd and soon thereafter served a cow there, yet this was not sufficient to transmit the disease to the healthy herd.

On March 3, 1902, I received, through the courtesy of Dr. S. T. Miller, two heifers from a badly diseased herd in Shelby County, Iowa. One of these heifers had been afflicted for about two weeks, and the other was in the very early stage of the disease. On March 8, 1902, two healthy heifers purchased from a nearby healthy herd were placed in a small enclosure with them. At the end of three months the healthy heifers had failed to acquire the disease. I have no facts which would tend to prove conclusively that the disease is contagious.

*Symptomatology.* (1) *Mild Cases.* In *females* the attention of a close observer is first attracted to the disease by a considerable swelling and reddening of the vulva. On closer examination it is found that this organ is painful on pressure, and, perhaps, shows an elevation of temperature. There are no constitutional symptoms. Within a day or two there will be noticed upon the skin at the inferior extremities of the labiæ vulvæ one or more spots varying from one-eighth to one-half inch in size, which show a whitening and opacity of the epidermis. At these points shallow ulcers very soon develop. The ulcers are irregular in contour; there is absence of induration at the border, and the floor is either covered with a thin layer of serous, serofibrinous, or puriform exudate, or with a scab. The covering is easily removed, and upon removal the ulcer has a bright red appearance and bleeds readily upon being manipulated. The ulcers increase rapidly in size. The floor of well-developed ulcers shows small elevations which appear like granulations, but are really islands of tissue which have proven more resistant to the necrotic process. The usual seat of the ulceration is at or near the inferior commissure of the vulva, but it may be localized higher up on the vulva or even upon the anus. In case an ulcer is located close to the mucocutaneous border of the vulva and extends so deeply as to pass through the skin, its edge may encroach upon the mucous membrane of the vulva; otherwise the vulvar mucosa remains intact. There is no itching, no secretion from the vaginal or the vulvar mucous membrane, no discernible discomfort, and no constitutional disturbance. The disease is purely local, and is mild in its manifestations. In *males* the trouble is at times localized upon the anus and the skin contiguous thereto, but is more frequently found upon the folds of skin passing from the root of the tail toward the sides of the anus. The features of the ulceration here are the same as in the case of the related structures of the female.

(2) *Severe Cases.* Ulcerative ano-vulvitis may assume a very grave character, producing extensive necrosis of the tissues, and in the worst cases leading to the death of the animal. In *females* there is a considerable number of cases in which the ulcerative process leads to the loss of the lower portion of the lips of the vulva, leaving only a large, angry-looking, raw surface to mark their place. The ulcer in these cases is phagedenic in nature, bringing about a uniform and rapidly advancing superficial necrosis with sloughing of the necrotic tissues at a correspondingly rapid pace. No large masses of necrotic tissue remain to become putrid, but as fast as the tissue becomes devitalized it is cast off. Thus the process, no matter how much the body is invaded, always remains superficial. It is always a true ulcerative process. The necrosis is confined to the surface. In cases of this degree of severity the affected parts emit a very repulsive odor of putrefaction.

The animal loses flesh, is stunted in growth if a young, growing animal; the coat is roughened, and there is general unthriftiness. In a few cases the entire vulva and anus undergo disintegration. One yearling heifer which I examined in January, 1900, showed loss of all the tissues between the sacrum, a line about two inches inferior to the vulva, and the ischial tuberosities. The loss of tissue extended inward as far as the ano-rectal and the vulva-vaginal boundary. A very large, foul-looking cavity was thus produced. The odor was very repulsive. Still, the ulceration was superficial, the dead tissue being in a thin layer. This animal had been lying for several days and kept the recumbent position in spite of urging; refused to eat; had lost much flesh; its coat was rough; was constipated and the urine was retained; it strained at short intervals and persistently, though it did not succeed in passing any feces or urine. Its condition was deplorable. As recovery seemed out of the question, and, as the resulting deformity of the parts would have rendered the animal useless if healing could have been induced, the heifer was killed in order that an autopsy might be made. I have reports of eight other cases which were nearly if not quite as badly affected as this. Three of these died of the disease, and the others recovered. One of the three that died was a cow five years old, and the other two were heifers eight to ten months of age.

Animals in which the disease makes such alarming progress stand about with the back arched; lose flesh rapidly; become rough in the coat; are constipated; eat sparingly; emit a very offensive odor, and evidently suffer much discomfort. The two heifer calves spoken of above which died did not suffer from decubitus. They were able to walk about in the evening, but were found dead the next morning.

In *males*, so far as I have been able to learn, the disease does not assume the very grave form seen in females. The invasion is in some cases quite extensive about the anus and root of the tail. In the case of a steer, ten months old, which I inspected there was marked ulceration around the root of the tail, especially on the dorsal surface and extending forward over the last two sacral vertebræ. The destruction of tissue extended to the bone, and there seemed to be imminent danger that the tail would be separated from the body. I have not learned of any deaths in males from this disease.

*Course.* It must be admitted that, as a rule, the course of ulcerative ano-vulvitis is rather mild, even when no treatment is applied. I have a record of one herd which I inspected, nearly all the individuals in which were afflicted; yet, although they were not favored with any treatment, they all made good recovery, without deformity. The disease, however, assumed a chronic course, as it existed in the herd for about five months and did not disappear until the cattle had been put upon pasture in the spring. The disease, once started, will get a foothold upon all or nearly all the individuals in the herd in an incredibly short time, say in ten days or two weeks at most. Treatment cuts short the progress of the disease. Most cases will be cured in two weeks to a month. Severe cases yield much less promptly. One of the fatal cases in which the necrosis was very destructive to the tissues ran a very short course, the animal having died of the disease within two weeks after its onset. The heifer referred to above as having been killed for autopsy, and in which the destruction of tissue was very great, is an example of an acute form of the disease, the course in this case not having been over three weeks. Cases in which extensive necrosis occurs within two or three weeks are by no means seldom encountered.

The period of incubation, using this term with reserve in this connection, is very short. In one herd that I inspected a healthy cow for which the owner had traded became affected within a week after being put into a diseased herd. Two other cows brought into this diseased herd at another time also became diseased within a week. In another herd which came under my notice, two heifers about eight months of age, which had been brought into the diseased herd in a healthy condition ten days before my inspection, I found to be in the early stages of the disease. In still another case a car-load of fat cattle which had been affected were taken out of the yard for shipment, and twenty-five head of cows and steers were at once put into the yard. Within three weeks nearly all of them had contracted the disease.

The course of the disease in a six-month-old heifer which I had under observation was as follows: On March 3, 1902, two weeks after the onset of the disease, the lower part of the vulva was found destroyed by ulceration, leaving a raw surface about three inches in diameter. March 12th it was noticed that granulation was well established, and that the ulcer was diminished in area by one-half and covered by a dry scab. March 16th further improvement was noted. March 22d the heifer was practically well, a scab about one-third of an inch in diameter being the only evidence of disease apart from the deformity resulting from the loss of tissue.

The course in a grade heifer eight months old which I had under observation was as follows: March 3, 1902, this heifer showed on the right lip of the vulva near the inferior commissure a shallow ulcer three-fourths of an inch in diameter, and on the left lip a little higher up an ulcer of about half this size. March 7th this heifer showed marked improvement. March 12th is nearly well. March 16th has entirely recovered. Neither of these heifers received any treatment except a change of locality.

*Pathological Anatomy. Macroscopy.* In all cases there is at the inception of the attack tumefaction and congestion of the vulva or other parts affected. In *mild* cases ulcers appear on the labiæ vulvæ, most commonly at or near the lower commissure, or about the anus of females, or upon the anus or the folds of skin at the base of the tail in males. In ulcers of most recent development only the epidermis and the upper portion of the corium have undergone the destructive process, but soon the entire thickness of the corium breaks down and is sloughed off, leaving an ulcer of considerable depth. The ulcers are in a few cases nearly free of exudate and present a reddish floor, but are in most cases surmounted by a serous, seropurulent, purulent or scaly covering. This exudate is easily removed, and when removed there is presented an angry, red, ulcerating surface which bleeds readily upon manipulation or scraping. These ulcers are irregular in contour, and their borders are not indurated. Upon the floor of the deeper ulcers there may be seen islands of tissue which have proven to a certain degree resistant to the necrotic process. In *severe* cases the affected parts are involved in an extensive disintegrating process which rapidly invades the tissues. The entire anus and vulva and surrounding tissues for several inches in every direction may be destroyed. The surface of the enormous ulcer thus produced is covered by a layer of exudate and necrotic tissue. This layer is, however, in all cases which I have observed surprisingly thin. Instead of having a thick stratum of gangrenous tissue separated from the healthy tissue by a well-defined line of demarcation, as one might expect, there is this thin stratum of exudate without the presence of any distinct line of demarcation. The process of necrosis is at all times a superficial one. The dead tissue is cast off as soon as death takes place. The process is in the truest sense an ulcerative one. At some places upon the surface the tissues are frayed out, presenting a large number of filamentous projections one-half inch or more in length. When the necrotic débris is removed there is revealed a highly inflamed surface directly underneath. One needs to cut only a short distance—not over one-half inch at most—into this tissue in order to reach apparently healthy tissue.

In one animal, a yearling heifer, referred to above as being very badly affected, upon which I had an opportunity to make an autopsy, I was able to study the pathological conditions quite carefully. The external lesions were such as I have just described. The general condition of the body was nearly normal. The only departure from normal that was distinctly visible was a serous infiltration of the lymphatic glands, especially in the posterior part of the body. These glands were much enlarged, quite succulent and grayish in color, having lost the pinkish tinge common to healthy lymphatic glands. There was no indication of suppuration or disintegration of any sort in the lymphatic glands.

*Microscopy.* There is nothing especially characteristic about the microscopic changes in the tissues. These changes are such as are common to inflammatory conditions on surfaces in which the necrotic process predominates over the reparative process. In a general way it may be said that there is at the surface a stratum of necrotic tissue elements, underneath which is a stratum in which an active inflammatory process is going on, and which gradually merges into healthy tissue below.

At the surface edge of a section made through the floor of an ulcer and at right angles to the floor is a thin layer of detritus consisting of fragmented nuclei, broken-down cell contents which have undergone liquefaction and subsequent desiccation, and a liberal amount of fibrin of the fibrillar variety. Below this is a layer of dense cellular infiltration in which the cells are chiefly polymorphonuclear and small mononuclear leucocytes. The presence of the large number of polymorphonuclear leucocytes is in harmony with the clinical aspect of rapid ulceration. In the thinnest sections the two varieties of cells mentioned are packed so closely together as to obscure or at least to make uncertain the identification of any other varieties of cells which might be present. Below this is a deeper layer of round-cell infiltration which gradually merges into the healthy tissue below. This layer is freer from fibrin than the other layers and is free of detritus. In double staining more of the nuclear and less of the contrast stain is taken. The field is much clearer and more transparent. The walls of the bloodvessels in this stratum show cellular proliferation which results in considerable thickening of their walls. The lumen of the vessels and the perivascular spaces are filled with blood corpuscles of which the red ones greatly predominate. Accumulations of red blood cells partly broken down are seen at other places without any special reference to the vessels. Occasionally there can be seen newly formed capillaries. A good deal of fibrillar fibrin is found scattered throughout this layer. Plasma cells, polymorphonuclear leucocytes, transitional leucocytes, lymphocytes, and fibroblasts are met with on every hand in this stratum. There is marked serous infiltration between the trabeculæ of connective tissue. This is most prominent in cases where the ulceration has not advanced very far.

There are no well-defined lines of separation between the three strata just described. They merge gradually into one another. The predominant character of the one gives way insensibly to the predominant character of the other.

In one case I was enabled to study a small ulcer which had at its greatest depth but very little more than passed through the cutis. At the edge of the ulcer the integrity of the skin was preserved, there being no demonstrable lesion in it. Approaching toward the centre of the ulcer the first noticeable change was a necrosis of the epithelium of the epidermis, shown by a sort of melting together of the cells and the failure of their nuclei to take the stain. At this point there is no especial change in the corium. Still farther toward the centre of the ulcer round-cell infiltration and chromatolysis are encountered. These, of course, involve the corium. Even at this point the general outline of the epidermis is preserved, it being stretched, parchment-like, over the infiltrated corium beneath, although the morphology of the cells is impaired. Finally one comes to a point where the skin is entirely lost, and there is presented a picture such as already described elsewhere.

I have been able to study sections of tissue from animals suffering with this disease in all its stages. Some of these tissues were obtained at autopsy, while others were removed from the living animal at the time of treatment. The tissues were fixed in formaldehyde and embedded in celloidin. The stains used were Van Gieson's, the double stain of hematoxylin and eosin, the double stain of eosin and methylene blue, and Weigert's fibrin stain.

*Diagnosis.* The diagnosis of external ulcerative ano-vulvitis of cattle offers no difficulties. There is no other disease with which there is much likelihood that it will be confounded. The objective signs are sufficient ground upon which to base a diagnosis. The early evidences of the disease are apt to be overlooked. If there is an unusual accumulation of filth having the appearance of a small scab about the lower commissure of the vulva, or about the anus, or if these parts are swollen and reddened, there is reason to suspect the presence of the disease, and the animal should at once be properly secured and thoroughly examined.

*Differential Diagnosis.* (1) *Eczema.* Eczema of the skin of the anus or vulva may present naked-eye features similar to those of the early stages of ulcerative ano-vulvitis, but the presence of itching in eczema and its absence in this disease would probably serve to distinguish them from each other. Eczema is characterized by vesicles and by a more diffuse distribution. Moreover, it does not extend deeply into the tissues. The more severe forms of ulcerative ano-vulvitis could not be confounded with eczema.

(2) *Vesicular Exanthema of Cattle.* The absence of participation of the vulvar and the vaginal mucous membrane in the pathological process, the failure to establish any relation with coitus, the absence of involvement of the male genital organs, the extensive necrosis in bad cases of ulcerative ano-vulvitis would be sufficient to preclude the possibility of mistaking one of these diseases for the other.

*Prognosis.* As a rule, this may be considered favorable, even without treatment. There are some cases, however, which will run rapidly to a fatal termination unless properly treated. Treatment cuts short the course of the disease, and even severe cases, with few exceptions, yield well to treatment. The mortality in the outbreaks which have come under my observation has been about 2 per cent. There has been no mortality in cases which have been treated. Complete recovery, absence of deformity, and restoration of heifers to full usefulness are dependent upon treatment early in the disease in each individual case. If neglected any case may very soon get beyond control.

*Treatment.* This may be curative and probably preventive, although our ignorance as to the cause renders preventive treatment problematical. There is some indication that the causative factor is an infectious material derived from yards which have grown filthy from prolonged use without sufficient attention to cleanliness. If this be so, the inference would be that the disease can be prevented by thoroughly cleaning the yards, by ploughing and hauling to the fields for fertilizer several inches of the surface ground and replacing it with fresh earth, or, better, with gravel or brick pavement. The manurial value of the surface ground would, I believe, be sufficient to compensate for the labor of removing it. If an earth or gravel bottom is maintained it should always be kept well bedded with clean straw. Cattle yards are, as a rule, too large. If their size was diminished they could the more readily be kept clean.

Curative treatment has been followed with good success. The underlying principles are disinfection and the application of some protective dressing. My experience leads me to believe that the removal of the affected herd to new and clean quarters as soon as the presence of the disease is determined would be a very valuable part of the curative treatment, and would in the milder cases be all the treatment that is necessary.

Steddom[12] reports as follows, in reference to the treatment of an outbreak in Kansas: "The first appearance of this disease in the calves was noticed three weeks ago, and a week later treatment with medicine (nitrate of silver as a caustic, and creolin—5 per cent. solution—as a wash) was commenced. But one application of caustic and creolin has been given each animal. They are all rapidly recovering."

C. Miller[13] reports his treatment as follows: "Using carbolic acid as an antiseptic, I took hot water and cleansed the parts, tail included, thoroughly, after which I applied a strong solution of ordinary white lotion, using a piece of cotton in applying it to the parts. The tail where it came in contact with the vulva was covered with vaseline, also the parts of the vulva not involved in the ulcer. In those cases where the ulcer was small I touched with a pencil of silver nitrate, which had the effect of arresting the action of the micro-organism at once. In fact, all the ulcers in whatever stage responded to the former treatment and commenced healing slowly from the first application. These applications were made four consecutive mornings, when the healing process was so far advanced that further treatment was deemed unnecessary, and within ten days they were completely healed, with no traces of the disease except in those cases where the vulval tissues were so nearly completely destroyed before any application was made."

D. H. Miller[14] reports as follows upon his treatment: "I removed all the diseased tissue that I could with curved scissors. Of the fifteen treated I applied to one-third tincture of iodine, to another third was applied a strong solution of corrosive sublimate, and to the rest a strong solution of creolin. This treatment was repeated twice. All recovered very rapidly. One treatment appeared to be as good as another. There has never been any recurrence of the trouble in the two years since the outbreak."

S. T. Miller[15] reports as follows upon his method of treatment: "I used for treatment a wash of a strong solution of mercury bichloride to cleanse the parts, after which I applied an ointment made up as follows: Iodoform, 20 grains; oil of eucalyptus, 40 minims; carbolic acid, 20 minims; petrolatum enough to make 2 ounces. This treatment effected a very speedy and permanent cure."

Miller does not state the strength of the corrosive sublimate solution used, but has since informed me that it was about 1 to 200.

L. A. Klein[16] reports as follows upon the treatment he pursued in the outbreak at Ames: "My treatment consisted of cleansing with creolin solution, cauterizing with nitrate of silver, followed by daily dressing with an ointment of acetate of lead 20 parts, tannic acid 10 parts, carbolic acid 5 parts, lard 65 parts. The case did well under this treatment. It was not very far advanced when I took hold of it."

J. Nicholson[17] informs me that in a herd he was called upon to treat for ulcerative ano-vulvitis he at first made use of a 5 per cent. solution of carbolic acid, applied by projecting it upon the parts with an ordinary piston syringe, and followed it by an application of boric acid. This was used about two weeks without good results. The treatment was then changed to creolin (Pearson), 1 to 40, applied by means of a syringe. The disease yielded quickly to this treatment. Only two applications were made except in three very aggravated cases, which had to be separated from one another and treated by creolin applications over a considerable period of time.

Although other measures doubtless do bring good results, I believe the best results are to be obtained by removal of necrotic tissue with the scissors or the curette, disinfection with some strong disinfecting solution such as referred to above, followed by the application of some such ointment as is formulated above. The advantages in the use of an ointment are that it keeps a curative agent constantly in contact with the affected area, prevents further contamination, and provides in a measure against the excoriating influence of the movements of the tail.

I am convinced also that removal of the entire herd to new, clean quarters, and the subsequent isolation of the severe cases will materially hasten the recovery.

*Terminations.* Mild cases make a complete recovery without any visible scar formation. In moderately severe ulceration, where healing takes place without extensive scar formation, the labiæ of the vulva are frequently left permanently thickened, indurated, and with diminished elasticity. In animals in which the ulceration has been extensive, if recovery occurs there results a very marked deformity of the vulva. I offer the following observations which I have made in this connection :

CASE I.—Grade Hereford heifer, eight months old. This heifer had suffered considerable ulceration of the vulva, but the healing took place without treatment within five weeks after the beginning of the attack. After healing had taken place I noticed that the orifice of the vulva was so small as to barely admit my little finger. Believing that this heifer would be useless for breeding purposes, I performed oöphorectomy upon her. I have seen her a number of times since and she is doing well.

CASE II.—Grade Aberdeen Angus heifer, one year old. This heifer suffered a very severe attack, the ulceration extending quite deeply, as far laterally as the tuberosities of the ischium, upward to the tail and downward to a line two inches below the vulva. Since healing there is scarcely any trace of the lips of the vulva, and the external orifice admits only the index finger. The anus is considerably shrunken by scar-formation. The growth of the animal has been much interfered with by the disease.

CASE III.—Grade shorthorn heifer, eight months old. Was quite badly affected. After healing only a small vestige of the labiæ remained. The orifice will admit only the forefinger. The animal has been much stunted in growth.

CASE IV.—Grade shorthorn heifer, eight months old, which had been seriously affected. Now there is almost no part of the lips of the vulva left. The vulvar orifice is only a slit about five-eighths of an inch long, and admits with difficulty the end of a lead pencil of ordinary size. The urine is passed with considerable force in a stream about the size of a large wheat straw. This heifer is much stunted in growth on account of the disease.

CASE V.—Grade heifer, eight months old. The lips of the vulva are tumefied, knotted, and much indurated. The vulvar orifice is about one and one-half inches long, and admits the index finger easily. The animal is considerably stunted by the disease.

CASE VI.—Grade shorthorn yearling heifer. Vulva tumefied and indurated. Orifice will admit two fingers. There is considerable scar-formation.

In heifers thus deformed their posterior aspect bears a close resemblance to that of a steer, and a careful examination is in some cases required in order to make sure of the sex of the animal.

D. H. Miller[18] reports that in the worst cases among the heifers in a herd which he treated the vulva was entirely destroyed, and when healing occurred under the influence of treatment only a small hole was left for the heifer to urinate through.

In the most malignant cases the necrosis continues to an extreme limit, causing an extensive loss of tissue until finally death results.

*Economic Importance.* This has, in a large measure, been indicated under the head of Terminations. It is readily seen that such widespread loss of tissue with consequent deformity and contraction of the outlet of the genital canal would unfit a heifer or cow for breeding purposes. She would receive the service of the bull with difficulty if at all. But, even if service of the bull would be possible, or, if impregnation should be accomplished by some artificial means, still there would remain an obstacle to parturition which would, to say the least, render breeding impracticable. The only logical recourse is to submit such an animal to oöphorectomy. This would be little if any loss in case of a grade animal or a pure-bred one of small intrinsic value, but the loss would be very serious if a valuable pure-bred heifer or cow should be involved.

The loss through fatalities is not to be overlooked in the consideration of the economic importance of the disease. If we add to these factors the loss through poor nutrition, stunting in growth, trouble and expense of treatment and discouragement to the owner, there is in the aggregate enough economic loss to give ulcerative ano-vulvitis a rank of considerable importance in the catalogue of diseases which afflict our herds.

The owner of eighty head of cattle, about two-thirds of which were affected, and two of which died, estimated his loss on account of the invasion of the disease at $300. Another farmer sold a car-load of imperfectly fattened cattle because they became affected. He lost by having deficient weight and by a subsequent advance in the price of fat cattle per pound.

### Bibliography.

1. American Veterinary Review, vol. xxii. p. 70.
2. Journal of Comparative Medicine and Veterinary Archives, vol. xxii. pp. 89-92.
3. American Veterinary Review, vol. xxii. p. 70.
4. Journal of Comparative Medicine and Veterinary Archives, vol. xxii. pp. 89-92.
5. Fifteenth Annual Report, Bureau of Animal Industry, Washington, D. C., 1898, pp. 382–384.
6. Journal of Comparative Medicine and Veterinary Archives, vol. xxii. pp. 92–94, and American Veterinary Review, vol. xxiv. pp. 682–684.
7. Verbal Communication.
8. Breeders' Gazette, vol. xl. or xli.
9. American Veterinary Review, vol. xxvi. pp. 326–327, and Journal of Comparative Medicine and Veterinary Archives, vol. xxiii. pp. 285–286.
10. Verbal Communication.
11. Verbal Communication.
12. Fifteenth Annual Report, Bureau of Animal Industry, Washington, D. C., 1898, p. 383.
13. Journal of Comparative Medicine and Veterinary Archives, vol. xxii. pp. 91–92.
14. Personal Letter.
15. Journal of Comparative Medicine and Veterinary Archives, vol. xxiii. p. 286, and American Veterinary Review, vol. xxvi. p. 327.
16. Personal Letter.
17. Verbal Communication.
18. Personal Letter.

